# Yield of actionable variants from high-risk cancer gene panel testing in guideline-eligible and ineligible individuals

**DOI:** 10.3389/fgene.2026.1924232

**Published:** 2026-09-21

**Authors:** Sarah S. Lee, Jingwen Zhang, Sara L. Bristow, Susan Rojahn, Mia Mackall, Meenakshi Malhotra, Youbao Sha, Eileen Hoffman, Vivienne Souter, Minetta C. Liu, Adam C. ElNaggar, Bhavana Pothuri

**Affiliations:** 1 Department of Obstetrics and Gynecology, Division of Gynecologic Oncology, New York University Grossman School of Medicine, New York, NY, United States; 2 Natera, Inc., Austin, TX, United States

**Keywords:** breast cancer, colorectal cancer, endometrial cancer, hereditary cancer, multigene panel testing, ovarian cancer, pancreatic cancer, prostate cancer

## Abstract

**Introduction:**

Hereditary cancer testing can identify individuals with a significant risk of cancer, and provide an opportunity for early risk management, prevention, precision treatment strategies, and family cascade testing. Medical professional organizations have developed guidance on genetic testing criteria based on personal and family histories. However, many individuals with cancer-predisposing pathogenic germline variants remain unidentified. The objective of this study was to determine the utility of a multi-cancer, clinically actionable cancer syndrome gene panel in identifying pathogenic variants among individuals both meeting and not meeting national genetic testing criteria.

**Methods:**

This retrospective cohort study analyzed 153,767 unrelated consecutive women and men, unselected for personal or family cancer history, who were referred for genetic testing across multiple sites from June 2020 to August 2023. Positivity rates were evaluated and compared according to testing guideline eligibility and cancer site-specific recommended gene sets.

**Results:**

In a multi-cancer panel of 29 clinically actionable genes, pathogenic or likely pathogenic variants were identified in 6.5% of individuals meeting hereditary testing criteria and 4.8% of those not meeting criteria. Overall, 22% of individuals with positive findings would have been missed had guideline-informed testing criteria been used, suggesting substantial missed diagnostic and therapeutic opportunities. Gene-level analyses revealed higher positivity rates for *BRCA2* (met: 1.2%, not met: 0.6%), *BRCA1* (met: 1.0%, not met: 0.3%), and *ATM* (met: 0.7%, not met: 0.53%) among individuals meeting only hereditary breast and ovarian cancer criteria, and for *MSH2* (met: 0.9%, not met: 0.05%), and *MLH1* (met: 0.6%, not met: 0.04%), and *PMS2* (met: 0.8%, not met: 0.36%) among those meeting only Lynch syndrome criteria.

**Discussion:**

These findings indicate that strict testing criteria may miss 22% of individuals with an actionable hereditary cancer gene variant. The use of comprehensive multi-cancer panels, alongside broader eligibility criteria, can identify individuals with hereditary cancer syndromes and improve opportunities for targeted screening, clinical management, and cascade testing for relatives.

## Introduction

Hereditary cancer syndromes account for approximately 10% of all cancers, though this varies across cancer types (Esplin et al., 2022), with 5%–10% of breast cancers and 22%–25% of ovarian cancers attributable to inherited pathogenic or likely pathogenic variants (PVs) (Apostolou and Fostira, 2013; Pietragalla et al., 2020; Walsh et al., 2011). Population-based studies have demonstrated that individuals carrying inherited PVs in genes associated with these syndromes have a higher cancer risk than those without such variants (Breast Cancer Association Consortium et al., 2021; Hu et al., 2021). For example, while the lifetime risk of breast cancer in the general population is approximately 13%, this risk increases to more than 60% in individuals with a PV in *BRCA1* or *BRCA2,* the genes most commonly associated with hereditary breast and ovarian cancer syndrome (HBOC) (Daly et al., 2025; Institute of Medicine US and National Research Council US et al., 2005; Kuchenbaecker et al., 2017). Similarly, for ovarian cancer, *BRCA1* and *BRCA2* PVs increase the lifetime risk from <2% to 39%–58% and 13%–29%, respectively (Daly et al., 2025; Antoniou et al., 2003).

Mounting evidence has demonstrated that individuals with hereditary cancer syndromes benefit from clinical management informed by genetic testing (Chiba et al., 2016; Domchek et al., 2010; Robson et al., 2017; Schrag et al., 2000; Yadav et al., 2018). This includes risk-reducing surgery such as mastectomy or salpingo-oophorectomy in individuals with PVs in *BRCA1/2* (Domchek et al., 2010; Finch et al., 2014), consideration of risk-reducing hysterectomy or screening with endometrial biopsy in individuals with PVs in genes associated with Lynch syndrome (LS) (i.e., *MLH1*, *MSH2*, *MSH6*, *PMS2*, *EPCAM*) (Gupta et al., 2025), and molecularly targeted therapies such as PARP inhibitors or immune checkpoint inhibitors when cancer is diagnosed (Daly et al., 2025). Detection of inherited PVs in cancer-syndrome genes also enables prevention and treatment opportunities in relatives via cascade testing.

Given the established increased cancer risk and the potential for prevention and treatment, professional society guidelines provide recommendations on eligibility for hereditary cancer genetic testing and specify which genes should be tested based on personal and family cancer history (Daly et al., 2025; Gupta et al., 2025). However, many individuals with hereditary cancer syndromes do not undergo genetic testing until after a cancer diagnosis, even when their family history meets testing criteria (Lee et al., 2023), resulting in missed opportunities for risk-reducing surgery (Smith et al., 2021), as well as delayed diagnosis, treatment optimization, and cascade testing. Furthermore, prior studies report that 10%–48% of patients with a personal history of cancer harbor PVs in cancer-related genes that did not meet guideline-based testing criteria (Beitsch et al., 2019; Kwong et al., 2023; Mandelker et al., 2017; Samadder et al., 2021; Shore et al., 2023; Yang et al., 2018), and guideline-recommended gene sets may not encompass all hereditary cancer syndromes (Samadder et al., 2021; Heald et al., 2022). In this study, we evaluated the impact of hereditary cancer testing guidelines and gene panel composition on the PV rate in individuals. Specifically, we assessed the utility of a multi-cancer, clinically actionable hereditary cancer syndrome gene panel for identifying PVs among individuals who do and do not meet national guideline criteria for genetic testing.

## Materials and methods

### Study population

Consecutive individuals aged ≥18 years who underwent hereditary cancer testing of a minimum 29-gene panel at a single commercial laboratory between June 2020 and August 2023 (N = 193,890) were considered for inclusion in this study. After excluding blood relatives of any proband (i.e., the first person tested among blood relatives) in the cohort (N = 1,284), a final study population of unrelated individuals (N = 192,606) were included. Testing was ordered at the discretion of the referring provider. Provider-reported demographic and clinical information, including age at testing, sex, race and ethnicity, personal cancer history, and family cancer history were abstracted for analysis.

Individuals were classified as either meeting or not meeting germline testing criteria according to the National Comprehensive Cancer Network (NCCN) guideline frameworks for HBOC and LS at the time of data extraction (Supplementary Appendix 1) (National Comprehensive Cancer Network, 2023a; National Comprehensive Cancer Network, 2023b). Classification was completed using an internal algorithm that used clinician-provided information to determine if an individual meets any of the sets of criteria for the testing for HBOC or LS. The clinician-reported information used to classify each individual could vary, and individuals could be missing information and still be classified as meeting or not meeting criteria. Individuals with incomplete personal or family history that resulted in an inability to definitively be classified as meeting or not meeting testing criteria as listed in Supplementary Appendix 1 were excluded (N = 38,839).

### Genetic testing

The set of 29 genes included in the multi-cancer panel for this study was selected based on two criteria: (1) a documented increased risk for breast, ovarian, endometrial, prostate, pancreatic, and/or colorectal cancer when a PV is identified and (2) established actionable clinical management recommendations based on NCCN guidelines (Table 1). The 29 clinically actionable genes that were the focus of this study are subsets of larger commercially available multiplex gene panels consisting of 40, 53, or 81 genes (Supplementary Appendix 2) or may have been included in custom panels with up to 191 genes. The additional genes included in these commercially available panels are associated with an increased cancer risk; however, clinical management recommendations for these genes have not yet been established in current guidelines.

**TABLE 1:** Genes associated with hereditary cancer syndromes with clinical management implications according to NCCN guidelines.

Gene	Inheritance pattern	Associated cancers	Actionability
*APC*	AD	Colorectal	Colonoscopic surveillance; upper endoscopy/duodenal surveillance; colectomy depending on phenotype/burden
*ATM*	AD	Breast, prostate, pancreatic	Annual mammogram at 40; consider breast MRI starting at age 30–35 years; pancreatic screening; consider prostate screening at age 40 years
*AXIN2*	AD	Colorectal	Colonoscopy-based colorectal surveillance; syndrome-specific upper-GI surveillance may apply
*BARD1*	AD	Breast	Annual mammogram and consider breast MRI starting at age 40 years
*BMPR1A*	​	Colorectal	Colonoscopy-based colorectal surveillance
*BRCA1*	AD	Breast, ovarian, prostate, pancreatic	Annual breast MRI from age 25 years, annual mammogram + MRI from age 30 years, risk-reducing mastectomy discussion, ovarian risk-reduction guidance, also pancreatic screening guidance and prostate management
*BRCA2*	AD	Breast, ovarian, prostate, pancreatic	Annual breast MRI from age 25 years, annual mammogram + MRI from age 30 years, risk-reducing mastectomy discussion, ovarian risk-reduction guidance, also pancreatic screening guidance and prostate management
*BRIP1*	AD	Ovarian	Recommend RRSO starting at age 45–50 years
*CDH1*	AD	Breast	Annual mammogram + consider MRI from age 30 years; discuss RRM
*CDKN2A*	AD	Pancreatic	Pancreatic screening per PANC-A; dermatologic surveillance for melanoma risk
*CHEK2*	AD	Breast, prostate, colorectal	Annual mammogram at age 40 years; consider breast MRI at age 30–35 years; consider prostate screening at age 40 years. Low-risk variants such as Ile157Thr/Ser428Phe/Thr476Met are exceptions
*EPCAM*	AD	Ovarian, endometrial, prostate, colorectal, pancreatic	Lynch management; colonoscopy and syndrome-specific surveillance
*GREM1*	AD	Colorectal	Colonoscopy q1–2years starting at age 20–25 years
*MLH1*	AD	Ovarian, endometrial, prostate, colorectal, pancreatic	Colonoscopy q1–2years starting at age 20–25 years; consider aspirin; gynecologic and upper-GI surveillance/risk-reduction discussions
*MSH2*	AD	Ovarian, endometrial, prostate, colorectal, pancreatic	Lynch management; colonoscopy q1–2years starting age 20–25 years, aspirin consideration, other surveillance based on syndrome/family history
*MSH6*	AD	Ovarian, endometrial, prostate, colorectal, pancreatic	Lynch management; colonoscopy and syndrome-specific surveillance
*MUTYH* ^a^	AR	Colorectal	MAP surveillance with colonoscopy
*NF1*	AD	Breast	Annual mammogram starting at age 30 years; consider breast MRI at ages 30–50 years; specialist referral for NF1-associated tumors
*NTHL1* ^a^	AR	Colorectal	Colonoscopic surveillance for biallelic disease
*PALB2*	AD	Breast, ovarian, pancreatic	Annual mammogram + breast MRI at age 30 years; discuss RRM; consider RRSO at age 45–50 years; pancreatic screening if family history; consider prostate screening at age 40 years
*PMS2*	AD	Endometrial, colorectal	Lynch management; colonoscopy and individualized extracolonic surveillance
*POLD1*	AD	Colorectal	Colonoscopy q1–2years starting at age 20–25 years
*POLE*	AD	Colorectal	Colonoscopy q1–2years starting at age 20–25 years
*PTEN*	AD	Breast, endometrial, colorectal	Breast surveillance plus thyroid, colorectal, endometrial, and renal surveillance
*RAD51C*	AD	Breast, ovarian	Annual mammogram + consider MRI at age 40 years; recommend RRSO at age 45–50 years
*RAD51D*	AD	Breast, ovarian	Annual mammogram + consider MRI at age 40 years; recommend RRSO at age 45–50 years
*SMAD4*	AD	Colorectal	Colonoscopy with polypectomy
*STK11*	AD	Breast, endometrial, colorectal, pancreatic	Colonoscopy with polypectomy, Annual mammogram + consider MRI at age 30 years; recommend RRSO at age 45–50 years; pancreatic screening at age 30–35 years
*TP53*	AD	Breast, prostate	Li-Fraumeni management; breast surveillance, whole-body/organ-specific surveillance, prostate screening at age 40 years, pancreatic screening if family history

^a^ Only individuals with biallelic pathogenic/likely pathogenic germline variants were considered to have a positive result.

DNA extracted from whole blood was subjected to next-generation sequencing (NGS) (Empower™, Natera, Inc., in collaboration with Baylor Genetics). The targeted regions of the genes associated with hereditary cancer syndromes were enriched using a capture-based method and sequenced (Illumina, Inc.). The variants detected in exons and within 20 bp of the exon/intron boundary were reported unless otherwise specified. Read-depth analysis was used to detect copy number variants (CNV) in genes. Gene-specific long-range polymerase chain reaction and Sanger sequencing confirmed positive results from certain genes or regions with highly homologous sequences across the genome. Multiplex ligation-dependent probe amplification (MLPA), PCR-based methods, and/or array comparative genomic hybridization (aCGH) were used to confirm CNVs involving the genes in the test. Variants were classified per the joint variant interpretation guidelines from the American College of Medical Genetics and Genomics and the Association for Molecular Pathology variant interpretation guidelines, internal protocols, and our current understanding of genes and variants at the time of reporting (Richards et al., 2015).

### Analysis

Summary statistics were used to describe the individual characteristics of the final cohort (met, not met NCCN testing criteria) and those excluded due to incomplete personal or family history.

Genetic test results from the 29-gene, clinically actionable panel were analyzed in two ways. First, the overall germline PV rate (i.e., the proportion of individuals with a PV in one or more of the 29 clinically actionable genes) was determined. Second, the germline PV rate was calculated for each of the 29 genes (i.e., the number of individuals with a PV in each gene). In all analyses of the PV rate, a single PV in genes except *MUTYH* and *NTHL1* was considered a positive finding. For *MUTYH* and *NTHL1*, a positive result required biallelic PVs.

In the primary analysis, individuals were categorized into two groups based on whether they met the testing criteria in at least one of the NCCN hereditary cancer risk guidelines (HBOC and/or LS) or did not meet any of the criteria (i.e., met vs. not met). In the secondary analysis, individuals who met at least one NCCN testing criterion were further subdivided into one of three mutually exclusive groups: (1) those who only met the testing criteria for breast, ovarian, pancreatic, and prostate cancers (HBOC guidelines), (2) those who only met the testing criteria for colorectal and endometrial cancer (LS guidelines), and (3) those who met both testing criteria.

In all analyses, continuous variables (i.e., age) were compared using the Wilcoxon test and categorical variables were compared using the Pearson chi square test. In the primary analyses, p-values were adjusted for multiple hypothesis testing (p < 0.00166). Statistical results for the secondary analysis were not adjusted (p < 0.05). Logistic regression analysis adjusted for age at the time of testing and for self-reported race and ethnicity was conducted and odds ratios (ORs) with 95% CIs were reported.

The percentage of individuals with PVs in genes included in the commercially available larger multiplex gene panels (40 genes, 53 genes, or 81 genes), excluding the 29-gene panel, was determined across all individuals.

All analyses were performed using R version 4.4.0 or higher (R Core Team, 2025).

## Results

### Study cohort characteristics

Among the 192,606 individuals eligible for study inclusion, 153,767 had sufficient clinical information to determine whether NCCN testing criteria were met (72.1%, 110,905/153,767) or not met (27.8%, 42,817/153,767) and were included in the final dataset for analysis (Table 2). The final cohort was mostly female (95.1%, 146,198/13,767) and had a diverse makeup of self-reported race and ethnicity, with less than half of the group being white (48.1%, 73,977/153,767). Most individuals were referred for testing through OB/GYN and midwifery providers (met: N = 77,286, 69.7%; not met: N = 27,636, 64.5%). While there were no differences in sex between those who met or did not meet testing criteria (female, met: 94.9%, not met: 95.1%, p = 0.91), differences were observed in age at testing (median [range], met: 44.3 years [34.8–56.0], not met: 46.3 years [36.0–58.3], p < 0.001) and in reported race and ethnicity (p < 0.001). Compared to individuals who did not meet testing criteria, individuals who met testing criteria had higher rates of reported personal history of cancer (16.9% vs. 13.9%, OR: 1.4 [95% CI: 1.36–1.45], p < 0.001) and family history of cancer (96.5% vs. 63.1%, OR: 25.1 [95% CI: 23.989–26.26], p < 0.001).

**TABLE 2:** Cohort characteristics.

Characteristic	Category	NCCN testing criteria met		p-value	OR (95% CI)
		None (N = 42,817)	At least one (N = 110,950)		
Age, years, median (Q1, Q3)	Years	46.3 (36.0, 58.3)	44.3 (34.9, 55.9)	<0.001	N/A
Sex, N (%)	Female	40,644 (94.9)	105,554 (95.1)	0.091	Ref.
​	Male	2196 (5.1)	5388 (4.9)		0.96 (0.91–1.01)
​	Unknown	4 (<0.01)	8 (<0.01)		N/A
Race/Ethnicity, N (%)	Ashkenazi Jewish	307 (0.7)	2452 (2.2)	<0.001	2.72 (2.42–3.08)
​	Asian	1228 (2.9)	2547 (2.3)		0.71 (0.66–0.76)
​	Black	5074 (11.9)	13,435 (12.1)		0.9 (0.87–0.94)
​	Hispanic	6504 (15.2)	14,441 (13.0)		0.76 (0.73–0.78
​	Other^a^	1716 (4.0)	3632 (3.3)		0.72 (0.68–0.76)
​	White	18,812 (43.9)	55,165 (49.7)		Ref.
​	Multiple	824 (1.9)	3281 (3.0)		1.36 (1.26–1.47)
​	Unknown	8352 (19.5)	15,997 (14.4)		0.65 (0.63–0.67)
Personal cancer history, N (%)	Yes	5947 (13.9)	18,797 (16.9)	<0.001	1.4 (1.36–1.45)
​	No	33,750 (78.8)	76,169 (68.7)		Ref.
​	Unknown	3120 (7.3)	15,984 (14.4)		2.27 (2.18–2.37)
Family cancer history, N (%)	Yes	27,018 (63.1)	107,103 (96.5)	<0.001	25.1 (23.98–26.26)
​	No	15,171 (35.4)	2397 (2.2)		Ref.
​	Unknown	628 (1.5)	1450 (1.3)		14.6 (13.17–16.23)

^a^ Other includes: French Canadian/Cajun, Mediterranean, Sephardic Jewish, Multiple, and Other.

Among the 38,839 individuals excluded because of incomplete personal or family history, there were statistical differences, though not clinically substantial, in median age, sex, or race/ethnicity compared to the final study population (Supplementary Table S1). As expected, compared to the final cohort, individuals who were excluded were most likely to have both an unknown personal history of cancer (excluded vs. final included: 32.3% vs. 12.4%, OR: 3.12 [95% CI: 3.03–3.20], p < 0.001) and family history of cancer (excluded vs. final included: 39.0% vs. 1.4%, OR: 122.61 [95% CI: 113.11–131.86], p < 0.001).

### Hereditary cancer testing results in a clinically actionable 29-gene panel

We calculated the proportion of individuals with a PV in at least one of the 29 clinically actionable genes to evaluate whether PV rates differed between those who met and those who did not meet NCCN testing criteria and compared the rates using ORs (95% CIs) adjusted for age and race/ethnicity based on logistic regression. Individuals who met testing criteria (6.5%, 7208/110,950) were slightly more likely to have a germline PV compared to those who did not meet testing criteria (4.8%, 2048/42,817) (OR: 1.32, 95% CI: 1.26–1.39, p < 0.000001) (Figure 1A). Among all 9256 individuals in the final cohort with a PV, 22.1% (N = 2048), or approximately one in five, would have been missed had the NCCN testing criteria been strictly followed (Figure 1B). Among individuals with PVs, most individuals had only one PV identified (met: 96.5%, 6956/7208; not met: 97.9%, 2005/2048). In the remaining individuals with PVs, those that met testing criteria had 2 (3.4%, 245/7248) or 3 PVs (0.1%, 7/7248), while those that did not meet testing criteria had 2 PVs (2.1%, 43/2048).

**FIGURE 1:**
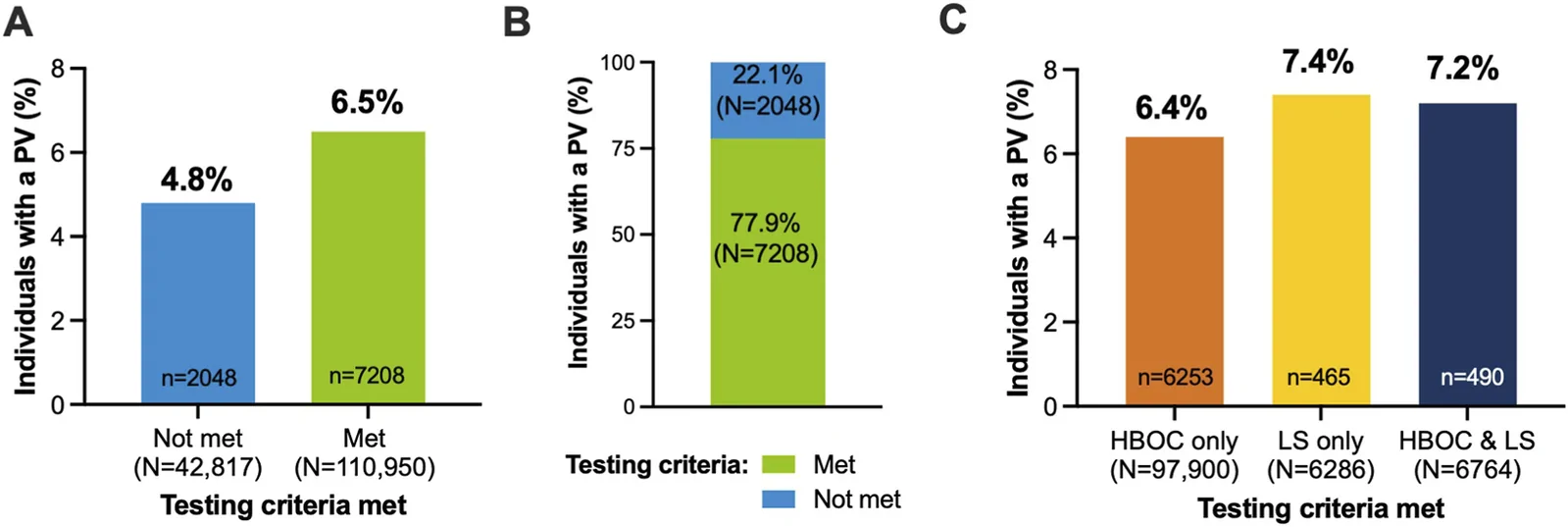
Hereditary cancer testing results from the 29-gene panel compared by NCCN criteria. **(A)** PV rates. Bar plots reporting the percentage of individuals with a germline PV in one of the 29 clinically actionable genes. The number of individuals in each cohort (“Not met” and “Met”) is indicated in parentheses under the bar chart. The number of individuals with a PV is reported at the bottom of each bar, and the percentage of individuals with a PV is reported above each bar. **(B)** Proportion of individuals that would have been missed if testing were done only in individuals who met testing criteria. Stacked bar chart showing the proportion and number of individuals with a PV based on whether they met (green) or did not meet (blue) testing criteria. The proportion and number of patients is reported inside each bar. **(C)** PV rates according to specific testing criteria. Bar plot reporting the percentage of individuals with a germline PV in one of the 29 clinically actionable genes. Individuals who met the testing criteria were included in this analysis and were grouped according to meeting only the HBOC testing criteria, only the LS testing criteria, or both. The number of individuals in each cohort (“HBOC,” “LS,” “HBOC & LS”) is indicated in parentheses under the bar chart. The number of individuals with a PV is reported at the bottom of each bar, and the proportion of individuals with a PV is reported above each bar. Abbreviations: HBOC, hereditary breast and ovarian cancer; LS, Lynch syndrome; PV, pathogenic or likely pathogenic variant.

To determine if the PV rate in the 29-gene multicancer panel varied among individuals who met testing criteria, the “met” testing criteria cohort was subdivided according to the testing criteria that were met: HBOC only (N = 97,900), LS only (N = 6286), or both (N = 6764). Across these three subgroups, the PV rate was 6.4% (N = 6253/97,900) for individuals who met the HBOC testing criteria, 7.4% (N = 465/6286) for those who met the LS testing criteria, and 7.2% (N = 490/6764) for those who met both criteria (likelihood ratio test, p < 0.01) (Figure 1C). In all three subgroups, most individuals had only one PV from the 29-gene panel (HBOC: 96.7%, N = 6045/6253; LS: 94.6%, N = 440/465; both: 96.1%, N = 440/490).

### Gene-level PV rates

Next, the PV rate for each of the 29 clinically actionable genes was compared between the met versus not met testing criteria groups to understand whether the observed difference in PV rate was due to specific genes. PV rates were statistically higher in individuals who met testing criteria compared to those who did not for *BRCA1* (OR: 2.65, 95% CI: 2.22–3.17, adjusted p < 0.000001), *MSH2* (OR: 2.42, 95% CI: 1.51–3.88, adjusted p < 0.001), *BRCA2* (OR: 1.77, 95% CI: 1.55–2.02, adjusted p < 0.000001), *BRIP1* (OR: 1.59, 95% CI: 1.24–2.04, adjusted p < 0.001), and *ATM* (OR: 1.30, 95% CI: 1.12–1.51, adjusted p < 0.001) (Figures 2A,B; Supplementary Table S2). No genes had PVs enriched in individuals who did not meet testing criteria.

**FIGURE 2:**
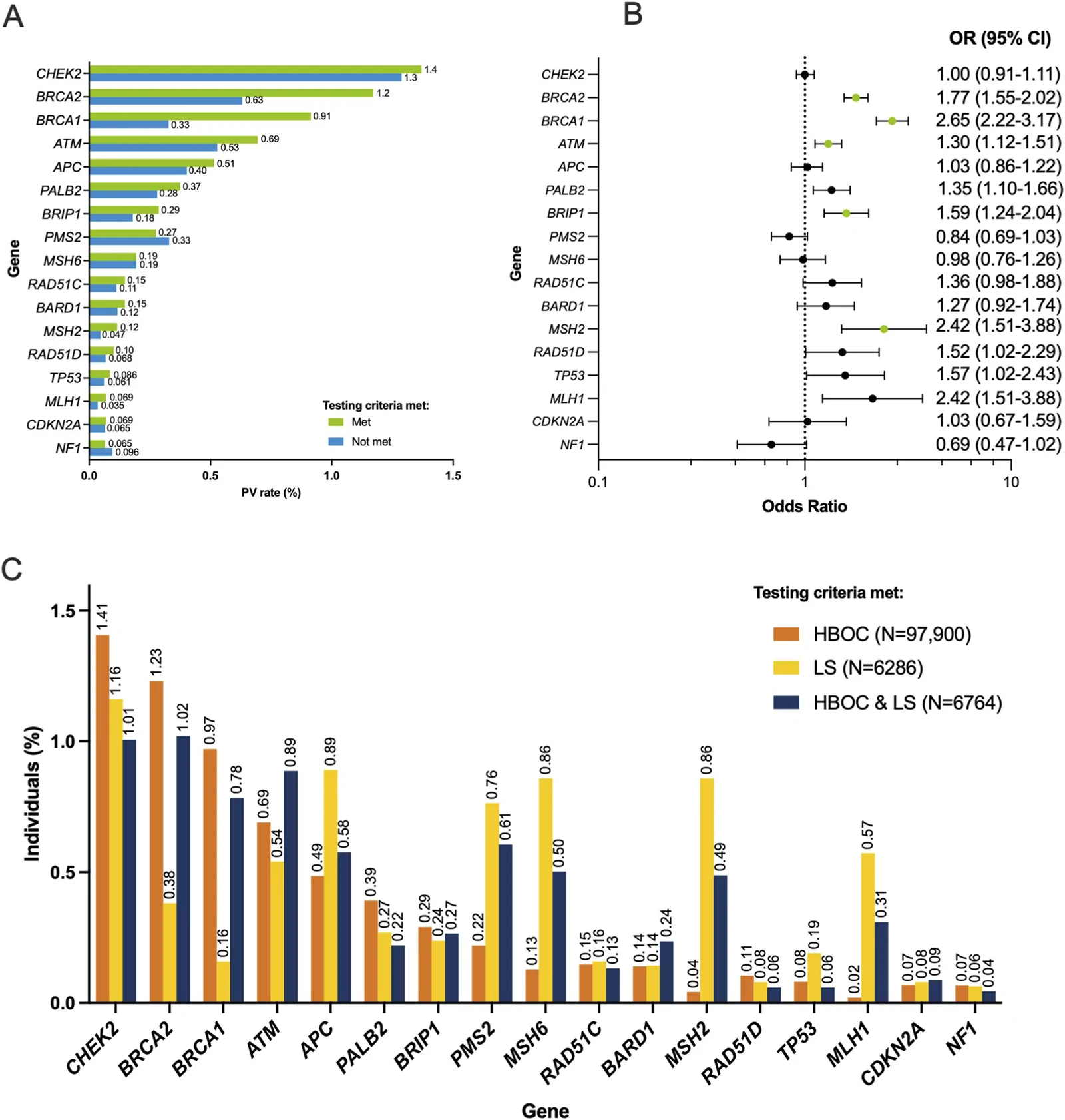
**(A)** Gene-level PV rates based on meeting NCCN testing criteria. Bar plot reporting the percent of individuals with a germline PV in each gene. **(B)** ORs associated with gene-level PV rates. Forest plot showing the ORs and 95% CIs associated with the differences in PV rates based on meeting testing criteria for each cancer-specific gene. ORs were adjusted for age, sex, and race and ethnicity. Green dots indicate that the PV rate was statistically higher in individuals who met testing criteria, whereas blue dots indicate that the PV rate was statistically higher in individuals who did not meet any testing criteria. **(C)** Gene-level PV rates based on meeting one or both NCCN testing criteria. Bar plot reporting the percent of individuals with a germline PV in each gene based on meeting one or both of the testing criteria. Genes with PV rates <0.05% in both criteria groups are not shown. All data for these genes and those not shown are reported in Supplementary Table S2.

To understand whether specific genes were driving PV rates based on which testing criteria were met, we calculated gene-level PV rates in individuals meeting HBOC criteria only, LS criteria only, or HBOC and LS criteria, and descriptively compared findings to individuals who met no testing criteria (i.e., “not met”). Among individuals who met HBOC testing criteria, PV rates were enriched in genes associated with breast and ovarian cancer compared to those who did not meet any testing criteria, including *BRCA2* (HBOC: 1.2%, not met: 0.6%) and *BRCA1* (HBOC: 1.0%, not met: 0.3%) (Figure 2C; Supplementary Table S2). Notably, PV rates were enriched in those who did not meet any testing criteria relative to those meeting HBOC testing criteria for two genes associated with LS: *PMS2* (HBOC: 0.2%, not met: 0.3%) and *MSH6* (HBOC: 0.1%, not met: 0.2%).

Conversely, compared to those who did not meet any testing criteria, individuals who met LS testing criteria had higher PV rates in genes associated with colorectal cancer and other LS-related genes, including *MSH2* (LS: 0.9%, not met: 0.05%) and *MLH1* (LS: 0.6%, not met: 0.04%) (Figure 2C; Supplementary Table S2). PV rates in breast and ovarian cancer risk genes were statistically lower in individuals meeting LS testing criteria compared to those meeting no testing criteria, including *BRCA2* (LS: 0.4%, not met: 0.6%) and *BRCA1* (LS: 0.2%, not met: 0.3%).

### Additional gene findings

Individuals in the study had additional provider-ordered genes, either as part of a standard panel comprising 40, 53, or 81 genes (Supplementary Appendix 2) or as a part of a custom provider-selected gene panel (up to 191 genes). Among all individuals, regardless of the clinician-ordered panel, 1.5% (1666/110,950) who met testing criteria had a PV in a gene outside the 29-gene panel (Figure 3). The rate of additional findings was 1.5% (622/42,817) among individuals who did not meet testing criteria. Among individuals meeting NCCN testing guidelines, the most frequent genes with a PV in the expanded panels were *CFTR* (7.8%; 10/129 individuals tested), *TYR* (6.5%; 3/46), and *FANCM* (4.26%; 2/47). Among individuals who did not meet guidelines, PVs were most frequent in *CFTR* (4.69%; 3/64), *TYR* (2.78%; 1/36), and *ERCC3* (2.78%; 1/36). For 110 additional genes tested in one of the standard gene panels, no PVs were identified.

**FIGURE 3:**
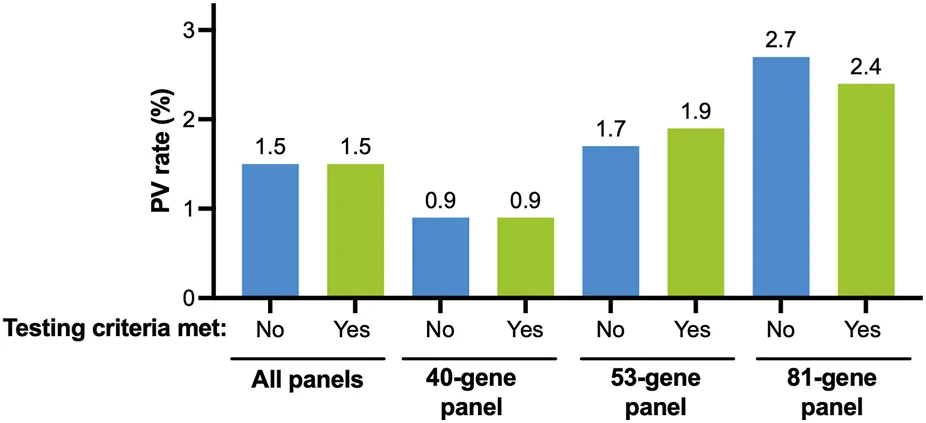
PV rates in provider-ordered genes outside of the clinically actionable, 29-gene panel. Bar plots depict the PV rates of additional findings in all individuals or by panel size. Individuals were divided into groups based on whether they met NCCN testing criteria.

To assess the effect of panel size on PV detection beyond the clinically actionable 29-gene panel, we calculated the PV rate among individuals who underwent 40-, 53-, or 81-gene panel testing. With increasing panel size, a higher proportion of individuals were found to carry a PV in an additional gene (40 genes: met, 0.9% [497/54,067], not met, 0.9% [190/20,932]; 53 genes: met 1.8% [686/37,521], not met 1.7% [235/14,474]; 81 genes: met 2.5% [457/19,071], not met 2.5% [184/7271]) (Figure 3). Notably, for each panel size, the rate of PVs in additional genes was comparable between individuals who met testing criteria and those who did not.

## Discussion

In this cohort of more than 150,000 individuals, we evaluated the prevalence of clinically actionable PVs according to NCCN testing eligibility. Although the detection rate of PVs in the multi-cancer, clinically actionable gene set was higher among individuals who met testing criteria than among those who did not (6.5% vs. 4.8%; OR: 1.32, 95% CI: 1.26–1.39), nearly one-quarter (22%) of individuals with a PV in these genes did not meet eligibility criteria and would have been overlooked had testing been restricted accordingly. These findings indicate that expanding the existing testing criteria could identify additional individuals at hereditary risk, thereby enabling preventative risk-reduction strategies, informing treatment decisions, and facilitating cascade testing in relatives.

The expansion of testing criteria raises the question of whether hereditary risk differs between individuals who do and do not meet eligibility requirements. At the level of individual genes, PV frequencies were consistently higher among individuals who met testing criteria; however, PVs that were common in eligible individuals were similarly prevalent among those who were ineligible. These data suggest that no single gene or subset of genes accounts for the observed differences in PV detection based on testing eligibility. Rather, the benefit of broader testing does not appear to be driven by gene-specific risk.

Overall, PV rates were comparable across eligibility sub-groups (HBOC only: 6.4%, LS only: 7.4%, both criteria: 7.2%). As expected, differences in the distribution of affected genes emerged in individuals meeting only the HBOC versus only the LS criteria, with the enrichment of genes associated with the respective cancer syndrome. For example, compared with individuals meeting only LS criteria, those meeting only HBOC criteria were 5 times more likely to have a PV in *BRCA1* (1.0% vs. 0.2%) and 3 times more likely to have a PV in *BRCA2* (1.2% vs. 0.4%). Conversely, PVs in LS-associated genes were more frequent among individuals meeting the LS testing criteria than among those meeting the HBOC testing criteria (*MSH2*: 0.9% vs. 0.04%, *PMS2*: 0.8% vs. 0.2%). These data demonstrate that testing criteria enrich genes associated with the relevant cancer syndromes. These findings support the use of multicancer gene panels with established clinical actionability, even among individuals who meet only a single set of testing criteria.

This study reports PV rates in a large cohort of individuals referred to a single commercial lab for hereditary cancer testing. The proportion of individuals with a PV who did not meet testing criteria (22%) was within the range reported in prior studies (10%–55%) (Beitsch et al., 2019; Kwong et al., 2023; Mandelker et al., 2017; Samadder et al., 2021; Yang et al., 2018; Bertelsen et al., 2019). In contrast to studies that have evaluated PV prevalence solely among individuals stratified by testing eligibility, this cohort included individuals both with and without a personal history of cancer. Moreover, the focus on 29 clinically actionable genes for assessing multiple cancer syndrome risks offers practical insight into genes most relevant to clinicians when selecting hereditary cancer testing panels. In two studies assessing PGV rates in patients with solid tumors, 55.5% and 53.2% of patients with PGVs did not meet testing criteria specific to each study (Mandelker et al., 2017; Samadder et al., 2021). These rates are likely much higher than our cohort because of the cohort selection. Our cohort was not required to have a personal history of cancer, leading to only 16% of individuals having a reported personal cancer history. In other studies that limited analysis to *BRCA1* and *BRCA2*, the PGV rates in these genes varied between 0.5% overall and 1.9%–3.2% depending on whether patients met or did not meet testing criteria, respectively (Yang et al., 2018; Manickam et al., 2018). When limiting our findings to *BRCA1* and *BRCA2*, our overall PGV rate was 1.8% (met: 2.2%, not met: 0.99%). The results from Manickam and colleagues represent a cohort that is close to unselected and population-based while the results from Yang and colleagues are from a cohort composed of patients mostly with a personal history of cancer. When considering germline testing results in the context of other studies, it is critical to consider the many factors that could explain the variation in the PV rate across studies, such as cohort selection, differences in genes examined, and the evolution of testing guidelines over time. For example, individuals included in this cohort were predominantly female, diverse racially/ethnically, and included both individuals with and without a personal cancer history, while these other studies have limited analysis to those with a personal history of cancer or could have other demographic differences.

An important question clinicians face is whether to select small, highly focused gene sets or large, comprehensive gene sets when testing an individual suspected of having a hereditary cancer syndrome. Multigene testing panels have continued to expand the number of genes with increasing technological advances and have expanded our collective knowledge of genes that increase cancer risks. Similar to previous reports (Henkel et al., 2025), we found that 1.5% of individuals in the cohort had a PV in the additional provider-ordered larger gene panels, suggesting that while the multi-cancer panel of 29 genes captures most individuals, additional individuals with hereditary cancer risk can be identified by larger gene sets.

The findings of this study have important implications for clinical decision-making related to referrals for hereditary cancer testing. Although testing guidelines provide a structured framework for identifying individuals who should be offered testing, clinical judgement and consideration of an individual’s unique circumstances may justify testing outside of established criteria. Family history remains a cornerstone of hereditary cancer risk assessment; however, such information may be unavailable, incomplete, or inaccurate for some individuals who harbor PVs in cancer predisposition genes. This limitation may be particularly relevant for individuals from populations with historically limited access to healthcare, for whom family history may not fully capture hereditary risk. In this cohort, a substantial proportion of individuals reported a family history of cancer (met: 96.5%, not met: 63.1%). Although detailed familial relationships were not available, these findings suggest that broadening how family history is defined or applied could help identify individuals who would otherwise not meet testing criteria. Moreover, while our PV rates were higher for a subset of genes among individuals who met testing criteria, this difference does not diminish the clinical importance of identifying and managing PVs in individuals who do not meet eligibility requirements.

The gene panel analyzed in this study was intentionally restricted to moderate- and high-penetrance genes with established implications for clinical management. Although variants identified beyond the core 29-gene panel may not currently meet criteria for clinical actionability, there are circumstances in which larger panels may be preferred by clinicians and patients. Emerging evidence may eventually support management changes for additional genes, and identification of a PV in these genes could prompt cascade testing of relatives, thereby raising awareness of increased cancer risk and enabling enhanced surveillance. Conversely, some clinicians and individuals may prefer smaller, more targeted gene panels to avoid more uncertain findings (i.e., variants of uncertain significance) (Rehm et al., 2023) or findings in genes for which no management recommendations exist. Ultimately, selection of hereditary cancer strategies should be individualized, with careful consideration of personal and family cancer history, gene penetrance, and potential benefits and limitations of broader versus more targeted testing approaches.

Within the US, there has been growing support for expanded hereditary cancer testing among individuals with solid tumors (Esplin et al., 2022), an approach that could identify a larger number of individuals with hereditary cancer syndromes and enable surveillance, preventive interventions, and treatment guidance. Broadening testing to a wider population could also reduce the burden on clinicians to determine testing eligibility, but barriers to testing would need to be addressed. Insurance coverage is not uniform across public and private payors, though a survey of providers indicated that costs are not a significant barrier because testing laboratories often aid in prior authorization requests and offer payment assistance programs (Lin et al., 2022). Future research evaluating the appropriateness of expanded testing, including cost-effectiveness analyses and comparisons of different gene panel sizes, would be valuable. The findings of this study support the use of a multicancer panel of clinically actionable genes to detect the majority of PVs. Additional prospective studies examining the clinical utility of such panels could further inform future implementation. Studies assessing clinician- and patient-reported outcomes could help elucidate the clinical and psychosocial impact of hereditary cancer testing results.

Several limitations should be considered when interpreting these findings. Because participants were referred for testing by their healthcare provider, the cohort does not reflect population-based testing. Individuals who pursued genetic testing in the current clinical environment may have an elevated underlying risk for hereditary cancer syndromes even if they did not meet testing criteria based on the information captured in this study. Although some individuals were classified as not meeting testing criteria, the provider may have relied on additional clinical details not available for analysis when ordering testing. Importantly, specific reasons for genetic testing from both providers and individuals were not captured in this study, and thus the accuracy of the algorithm used for classification could not be evaluated. An additional limitation is that 20% (38,839/192,606) of individuals tested during the study period were excluded due to incomplete personal or family history, which may have introduced bias. Differences were observed between excluded individuals and the final analytic cohort with respect to age of testing, sex, and self-reported race and ethnicity. Future prospective studies that assess PV rates in consecutively tested individuals with complete clinical information will be important to reduce exclusion due to missing data and better provide insights into the generalizability and applicability of the findings.

The findings of this study indicate that current hereditary cancer testing criteria fail to identify a meaningful number of individuals with clinically actionable PVs. This includes individuals who do not meet any testing criteria as well as those who meet only a single set of criteria and may therefore undergo more limited testing. In addition, a multicancer panel of clinically actionable genes across multiple cancer types captures the majority of germline PVs across individuals regardless of testing eligibility. Collectively, these results support both the expansion of hereditary cancer testing criteria and the broader use of multicancer gene panels.

## Data Availability

The datasets presented in this article are not readily available because the authors declare that all relevant non-proprietary data used to conduct the analyses are available within the article. To protect the privacy and confidentiality of the patients in this study, clinical data are not made publicly available in a repository but can be requested at any time from the corresponding author. All data shared will be de-identified. Requests to access the datasets should be directed to Adam ElNaggar, aelnaggar@natera.com.
