# Supplementary material for: Yield of actionable variants from high-risk cancer gene panel testing in guideline-eligible and ineligible individuals
Source: Front Genet. 2026 Sep 21;17:1924232. doi: 10.3389/fgene.2026.1924232 (PMC13634612; doi:10.3389/fgene.2026.1924232)
Supplement: Supplementary file 1 [file Supplementaryfile1.docx]

**Appendix 1 - Simplified NCCN Criteria**

**Individuals were determined to Meet Criteria for Hereditary Breast and Ovarian Cancer Syndrome testing (breast, ovarian, prostate, and pancreatic cancer) if:**

1. Individuals with a personal history of breast cancer diagnosed at ≤50 years of age
2. Individuals with a personal history of breast cancer and Ashkenazi Jewish ancestry
3. Individuals with a personal history of multiple primary breast cancers (synchronous or metachronous).
4. Individuals with a personal history of any rare cancer diagnosed at any age
   a. Rare cancers considered: Breast triple negative, ovarian, ovarian non-epithelial, male breast cancer, prostate, pancreatic
5. Individuals with a family history of breast cancer in a first- or second-degree relative who was diagnosed at ≤50 years of age
6. Individuals with a family history of breast triple negative, ovarian, ovarian non-epithelial cancer, or male breast cancer in a first- or second-degree relative who was diagnosed at any age
7. Individuals with a family history of prostate or pancreatic cancer in a first-degree relative who was diagnosed at any age
8. Individuals with Ashkenazi Jewish ancestry and a family history of breast cancer diagnosed in a first-, second-, or third-degree relative at any age
9. Individuals with a personal history of breast cancer at any age AND a family history of breast cancer diagnosed in ≥2 first- or second-degree relatives (or twice in same relative) on the same side of family
10. Individuals with a family member with a known pathogenic or likely pathogenic variant in an HBOC gene or gene combination

**Individuals were determined to Meet Criteria for Lynch Syndrome testing (colorectal and endometrial cancer) if:**

1. Individuals with a personal history of colorectal cancer diagnosed at any age
2. Individuals with a personal history of endometrial or colorectal cancer diagnosed ≤50 years of age
3. Individuals with a family history of endometrial or colorectal cancer in a first-degree relative who was diagnosed at ≤50 years of age
4. Individuals with a family member with a known pathogenic or likely pathogenic variant in any Lynch Syndrome gene, *APC*, or gene combination

Adapted from

Daly et al. Genetic/Familial High-Risk

Assessment: Breast, Ovarian, and Pancreatic, Version 3.2023.

and

Gupta et al. Genetic/Familial High-Risk Assessment: Colorectal, Version 1.2023
