# Supplementary material for: Yield of actionable variants from high-risk cancer gene panel testing in guideline-eligible and ineligible individuals
Source: Front Genet. 2026 Sep 21;17:1924232. doi: 10.3389/fgene.2026.1924232 (PMC13634612; doi:10.3389/fgene.2026.1924232)
Supplement: Supplementary file 2 [file Supplementaryfile2.docx]

**Appendix 2 Multiplex panel gene lists**

**40-Gene Panel**

*APC*, *ATM*, *AXIN2*, *BAP1*, *BARD1*, *BMPR1A*, *BRCA1*, *BRCA2*, *BRIP1*,*CDH1*, *CDK4*, *CDKN2A*, *CHEK2*, *EPCAM*, *GALNT12*, *GREM1*, *HOXB13*, *MEN1*, *MITF*, *MLH1*, *MSH2*, *MSH3*, *MSH6*, *MUTYH*, *NBN*, *NF1*, *NTHL1*, *PALB2*, *PMS2*, *POLD1*, *POLE*, *PTEN*, *RAD51C*, *RAD51D*, *RNF43*, *RPS20*, *SMAD4*, *STK11*, *TP53*, *VHL*

**53-Gene Panel**

*APC*, *ATM*, *AXIN2*, *BAP1*, *BARD1*, *BMPR1A*, *BRCA1*, *BRCA2*, *BRIP1*, *CDH1*, *CDK4*, *CDKN2A*, *CHEK2*, *CTNNA1*, *DICER1*, *EPCAM*, *GALNT12*, *GREM1*, *HOXB13*, *KIT*, *MEN1*, *MITF*, *MLH1*, *MRE11*, *MSH2*, *MSH3*, *MSH6*, *MUTYH*, *NBN*, *NF1*, *NTHL1*, *PALB2*, *PDGFRA*, *PMS2*, *POLD1*, *POLE*, *PTEN*, *RAD50*, *RAD51C*, *RAD51D*, *RNF43*, *RPS20*, *SDHA*, *SDHB*, *SDHC*, *SDHD*, *SMAD4*, *SMARCA4*, *STK11*, *TP53*, *TSC1*, *TSC2*, *VHL*

**81-Gene Panel**

*AIP*, *ALK*, *APC*, *ATM*, *AXIN2*, *BAP1*, *BARD1*, *BMPR1A*, *BRCA1*, *BRCA2*, *BRIP1*, *CDC73*, *CDH1*, *CDK4*, *CDKN1B*, *CDKN1C*, *CDKN2A*, *CEBPA*, *CHEK2*, *CYLD*, *DDX41*, *DICER1*, *EGFR*, *EPCAM*, *EXT1*, *EXT2*, *FH*, *FLCN*, *GATA2*, *GREM1*, *HOXB13*, *KIT*, *LZTR1*, *MAX*, *MEN1*, *MET*, *MITF*, *MLH1*, *MSH2*, *MSH3*, *MSH6*, *MUTYH*, *NBN*, *NF1*, *NF2*, *NTHL1*, *PALB2*, *PDGFRA*, *PHOX2B*, *PMS2*, *POLD1*, *POLE*, *POT1*, *PRKAR1A*, *PTCH1*, *PTEN*, *RAD51C*, *RAD51D*, *RB1*, *RET*, *RHBDF2*, *RUNX1*, *SDHA*, *SDHAF2*, *SDHB*, *SDHC*, *SDHD*, *SMAD4*, *SMARCA4*, *SMARCB1*, *SMARCE1*, *STK11*, *SUFU*, *TERC*, *TERT*, *TMEM127*, *TP53*, *TSC1*, *TSC2*, *VHL*, *WT1*
