# Supplementary material for: Yield of actionable variants from high-risk cancer gene panel testing in guideline-eligible and ineligible individuals
Source: Front Genet. 2026 Sep 21;17:1924232. doi: 10.3389/fgene.2026.1924232 (PMC13634612; doi:10.3389/fgene.2026.1924232)
Supplement: Supplementary file 3 [file Table1.docx]

**Supplemental Table 1. Characteristics of individuals with insufficient information for determining NCCN testing criteria**

|  |  | **Excluded cohort because of incomplete family/personal cancer history (N=38,839)** | **Final included cohort, all individuals**  **(N=153,767)** | **p** | **OR** |
| --- | --- | --- | --- | --- | --- |
| Age, median (Q1, Q3) | Years | 43.1 (33.8, 54.9) | 44.8 (35.2, 56.7) | <0.001 | N/A |
| Sex, n (%) | Female | 36,451 (93.9) | 146,198 (95.1) | <0.001 | Ref. |
|  | Male | 2,384 (6.1) | 7557 (4.9) |  | 1.27 (1.21-1.33) |
|  | Unknown | 4 (0.01) | 12 (0.01) |  | N/A |
| Race/Ethnicity, n (%)^a^ | Ashkenazi Jewish | 304 (0.8) | 2759 (1.8) | <0.001 | 0.53 (0.46-0.59) |
|  | Asian | 712 (1.8) | 3775 (2.5) |  | 0.9 (0.83-0.98) |
|  | Black | 6184 (15.9) | 18,509 (12.0) |  | 1.59 (1.54-1.65) |
|  | Hispanic | 5855 (15.1) | 20,945 (13.6) |  | 1.33 (1.29-1.38) |
|  | White | 15,511 (39.9) | 73,977 (48.1) |  | Ref. |
|  | Other | 1487 (3.8) | 5348 (3.5) |  | 1.33 (1.25-1.41) |
|  | Multiple | 772 (2.0) | 4105 (2.7) |  | 0.9 (0.83-0.97) |
|  | Unknown | 8014 (20.6) | 24,349 (15.8) |  | 1.57 (1.52-1.62) |
| Personal cancer history, n (%) | Yes | 3128 (8.1) | 24,744 (16.1) | <0.001 | 0.6 (0.58-0.62) |
|  | No | 23,167 (59.6) | 109,919 (71.5) |  | Ref. |
|  | Unknown | 12,544 (32.3) | 19,104 (12.4) |  | 3.12 (3.03-3.20) |
| Family cancer history, n (%) | Yes | 22,650 (58.3) | 134,121 (87.2) | <0.001 | 2.84 (2.67-3.03) |
|  | No | 1044 (2.7) | 17,568 (11.4) |  | Ref. |
|  | Unknown | 15,145 (39.2) | 2078 (1.4) |  | 122.61 (113.11-131.86) |

^a^Other includes: French Canadian/Cajun, Mediterranean, Sephardic Jewish, Multiple, and Other.
