# Supplementary material for: Yield of actionable variants from high-risk cancer gene panel testing in guideline-eligible and ineligible individuals
Source: Front Genet. 2026 Sep 21;17:1924232. doi: 10.3389/fgene.2026.1924232 (PMC13634612; doi:10.3389/fgene.2026.1924232)
Supplement: Supplementary file 4 [file Table2.docx]

**Supplemental Table 2. PV rates by gene**

|  | **Not met**  **(N=42,817)** | **Met**  **(N=110,950)** | | **HBOC**  **(N=97,900)** | | **LS**  **(N=6,286)** | | **Both**  **(N=6,764)** | |
| --- | --- | --- | --- | --- | --- | --- | --- | --- | --- |
| n (%) | **PV rate** | **PV rate** | **OR (95% CI), p-value^a^** | **PV rate** | **OR (95% CI)** | **PV rate** | **OR (95% CI)** | **PV rate** | **OR (95% CI)** |
| *CHEK2* | 551 (1.23) | 1,518 (1.37) | 1.00 (0.91-1.11)  Adj.p=0.938 | 1377 (1.4) | 1.04 (0.94-1.14)  p=0.498 | 73 (1.2) | 0.87 (0.68-1.11)  p=0.252 | 68 (1.0) | 0.71 (0.55-0.91)  p=0.00718 |
| *BRCA2* | 270 (0.63) | 1,298 (1.17) | 1.77 (1.55-2.02)  p<0.000001 | 1,205 (1.2) | 1.61 (1.23-2.10)  p<0.000001 | 24 (0.4) | 0.61 (0.40-0.93)  p=0.0206 | 69 (1.0) | 1.61 (1.23-2.10)  p=0.00045 |
| *BRCA1* | 140 (0.33) | 1,013 (0.91) | 2.65 (2.22-3.17)  p<0.000001 | 950 (1.0) | 2.79 (2.33-3.34)  p<0.000001 | 10 (0.2) | 0.51 (0.27-0.97)  p=0.0401 | 53 (0.8) | 2.50 (1.82-3.44)  p<0.000001 |
| *ATM* | 226 (0.53) | 770 (0.69) | 1.30 (1.12-1.51)  p=0.000636 | 676 (0.7) | 1.29 (1.11-1.50)  p=0.000961 | 34 (0.5) | 1.02 (0.71-1.46)  p=0.923 | 60 (0.9) | 1.64 (1.23-2.19)  p=0.000674 |
| *APC* | 172 (0.40) | 571 (0.51) | 1.03 (0.86-1.22)  p=0.753 | 476 (0.5) | 0.95 (0.79-1.13)  p=0.563 | 56 (0.9) | 2.17 (1.60-2.95)  p<0.000001 | 39 (0.6) | 1.22 (0.86-1.73)  p=0.276 |
| *PALB2* | 120 (0.28) | 416 (0.37) | 1.35 (1.10-1.66)  p=0.00404 | 384 (0.4) | 1.41 (1.15-1.74)  p=0.00101 | 17 (0.3) | 0.97 (0.58-1.61)  p=0.904 | 15 (0.2) | 0.80 (0.47-1.37)  p=0.416 |
| *BRIP1* | 77 (0.18) | 318 (0.29) | 1.61 (1.25-2.08)  p=0.000213 | 285 (0.3) | 1.61 (1.25-2.08)  p=0.000213 | 15 (0.2) | 1.34 (0.77-2.33)  p=0.303 | 18 (0.3) | 1.47 (0.88-2.45)  p=0.144 |
| *PMS2* | 141 (0.33) | 305 (0.27) | 0.84 (0.69-1.03)  p=0.0887 | 216 (0.2) | 0.67 (0.54-0.83)  p=0.000261 | 48 (0.8) | 2.32 (1.67-3.22)  p<0.000001 | 41 (0.6) | 1.81 (1.27-2.56)  p=0.000914 |
| *MSH6* | 83 (0.19) | 215 (0.19) | 0.98 (0.76-1.26)  p=0.8620 | 127 (0.1) | 0.65 (0.49-0.85)  p=0.002194 | 54 (1.0) | 4.41 (3.13-6.22)  p<0.000001 | 34 (0.5) | 2.49 (1.67-3.72)  p=0.000008 |
| *RAD51C* | 48 (0.11) | 164 (0.15) | 1.36 (0.98-1.88)  p=0.06186 | 145 (0.1) | 1.37 (0.99-1.91)  p=0.0584 | 10 (0.2) | 1.38 (0.70-2.72)  p=0.3597 | 9 (0.1) | 1.18 (0.58-2.40)  p=0.6529 |
| *BARD1* | 50 (0.12) | 163 (0.15) | 1.27 (0.92-1.74)  p=0.1436 | 138 (0.1) | 1.22 (0.88-1.69)  p=0.23376 | 9 (0.1) | 1.23 (0.60-2.50)  p=0.5719 | 16 (0.2) | 2.05 (1.16-3.60)  p=0.0130 |
| *MSH2* | 20 (0.046) | 128 (0.12) | 2.42 (1.51-3.88)  p=0.000252 | 41 (0.04) | 0.85 (0.50-1.45)  p=0.5523 | 54 (0.9) | 18.69 (11.17-31.28)  p<0.000001 | 33 (0.5) | 10.40 (5.95-18.16)  p<0.000001 |
| *RAD51D* | 29 (0.068) | 112 (0.10) | 1.52 (1.01-2.29)  p=0.0469 | 103 (0.1) | 1.58 (1.04-2.39)  p=0.0302 | 5 (0.08) | 1.38 (0.70-2.72)  p=0.3597 | 4 (0.06) | 0.90 (0.32-2.57)  p=0.8457 |
| *TP53* | 26 (0.061) | 95 (0.086) | 1.57 (1.02-2.43)  p=0.04196 | 79 (0.08) | 1.51 (0.97-2.37)  p=0.06844 | 12 (0.02) | 2.86 (1.44-5.68)  p=0.002636 | 4 (0.06) | 0.94 (0.33-2.70)  p=0.9121 |
| *MLH1* | 15 (0.035) | 77 (0.069) | 2.13 (1.22-3.71)  p=0.007738 | 20 (0.02) | 0.61 (0.31-1.20)  p=0.1511 | 36 (0.6) | 17.23 (9.41-31.54)  p<0.000001 | 21 (0.3) | 9.69 (4.98-18.86)  p<0.000001 |
| *CDKN2A* | 28 (0.065) | 77 (0.069) | 1.03 (0.67-1.59)  p=0.8878 | 66 (0.07) | 1.01 (0.64-1.57)  p=0.9783 | 5 (0.08) | 1.16 (0.45-3.02)  p=0.7542 | 6 (0.09) | 1.26 (0.52-3.04)  p=0.6142 |
| *NF1* | 41 (0.096) | 72 (0.065) | 0.69 (0.47-1.02)  p=0.0636 | 65 (0.07) | 0.72 (0.48-1.06)  p=0.0971 | 4 (0.06) | 0.64 (0.23-1.79)  p=0.3932 | 3 (0.04) | 0.45 (0.14-1.45)  p=0.1801 |
| *CDH1* | 16 (0.037) | 35 (0.032) | 0.81 (0.44-1.46)  p=0.4758 | 31 (0.03) | 0.80 (0.44-1.48)  p=0.4805 | 0 | 0 (0-inf)  p=0.9873 | 4 (0.06) | 1.56 (0.52-4.67)  p=0.4306 |
| *PTEN* | 3 (0.007) | 15 (0.014) | 1.91 (0.55-6.61)  p=0.3089 | 13 (0.01) | 1.85 (0.53-6.52)  p=0.3382 | 1 (0.02) | 2.42 (0.25-23.22)  p=0.4448 | 1 (0.01) | 2.30 (0.24-22.24)  p=0.4706 |
| *AXIN2* | 2 (0.005) | 15 (0.014) | 2.67 (0.61-11.75)  p=0.1943 | 11 (0.01) | 2.17 (0.48-9.85)  p=0.3175 | 3 (0.05) | 10.66 (1.78-63.97)  p=0.00965 | 1 (0.01) | 3.13 (0.28-34.71)  p=0.3528 |
| *MUTYH^b^* | 5 (0.012) | 14 (0.013) | 1.10 (0.39-3.08)  p=0.8533 | 7 (0.007) | 0.62 (0.19-1.96)  p=0.4119 | 6 (0.1) | 7.85 (2.39-25.81)  p=0.000691 | 1 (0.01) | 1.20 (0.14-10.30)  p=0.8681 |
| *GREM1* | 6 (0.014) | 9 (0.008) | 0.54 (0.19-1.54)  p=0.2491 | 5 (0.005) | 0.34 (0.10-1.12)  p=0.0753 | 1 (0.02) | 1.08 (0.13-8.96)  p=0.9451 | 3 (0.04) | 2.94 (0.73-11.81)  p=0.1283 |
| *EPCAM* | 1 (0.002) | 4 (0.004) | 1.62 (0.18-14.62)  p=0.6667 | 2 (0.002) | 0.91 (0.08-10.15)  p=0.9403 | 0 | 0 (0-inf)  p=0.9956 | 2 (0.03) | 13.44 (1.21-149.60)  p=0.03460 |
| *SMAD4* | 1 (0.002) | 4 (0.004) | 1.52 (0.17-13.61)  p=0.7106 | 2 (0.002) | 0.83 (0.08-9.22)  p=0.8817 | 1 (0.02) | 7.63 (0.47-123.17)  p=0.1522 | 1 (0.01) | 7.27 (0.45-117.03)  p=0.1619 |
| *STK11* | 1 (0.002) | 4 (0.004) | 1.53 (0.17-13.76)  p=0.7065 | 3 (0.003) | 1.31 (0.13-12.71)  p=0.8162 | 0 | 0 (0-inf)  p=0.9969 | 1 (0.01) | 5.65 (0.35-90.57)  p=0.2214 |
| *BMPR1A* | 2 (0.005) | 3 (0.003) | 0.56 (0.09-3.36)  p=0.5219 | 1 (0.001) | 0.21 (0.02-2.32)  p=0.2015 | 1 (0.02) | 3.03 (0.27-33.42)  p=0.3660 | 1 (0.01) | 2.72 (0.25-30.04)  p=0.4148 |
| *POLD1* | 1 (0.002) | 1 (0.001) | 0.37 (0.02-5.96)  p=0.4804 | 0 | 0 (0-inf)  p=0.9879 | 1 (0.02) | 6.33 (0.39-101.42)  p=0.1925 | 0 | 0 (0-inf)  p=0.9969 |
| *NTHL1^b^* | 0 | 0 | – | 0 | – | 0 | – | 0 | – |
| *POLE* | 0 | 0 | – | 0 | – | 0 | – | 0 | – |

^a^p<0.00166 were considered significant, accounting for multiple hypothesis testing. ^b^Only individuals with biallelic PVs were included.

Colored cells indicate that compared to individuals who did not meet any testing criteria, the PV rate is statistically higher in those who met any criteria.
